# Small-diameter titanium grade IV and titanium-zirconium implants in edentulous mandibles: five-year results from a double-blind, randomized controlled trial

**DOI:** 10.1186/s12903-015-0107-6

**Published:** 2015-10-12

**Authors:** Frauke Müller, Bilal Al-Nawas, Stefano Storelli, Marc Quirynen, Stefan Hicklin, Jose Castro-Laza, Renzo Bassetti, Martin Schimmel, On behalf of the Roxolid Study Group

**Affiliations:** 1 Division of Gerodontology and Removable Prosthodontics, University of Geneva, Geneva, Switzerland; 2 Johannes-Gutenberg University Mainz, Mainz, Germany; 3 University of Milan, Dental Clinic, San Paolo Hospital, Milan, Italy; 4 School of Dentistry, Catholic University Leuven, Leuven, Belgium; 5 University of Bern, School of Dental Medicine, Bern, Switzerland; 6 University of Regensburg Clinic, Regensburg, Germany; 7 Cantonal Hospital Lucerne, Clinic for Oral and Maxillofacial Surgery, Lucerne, Switzerland

**Keywords:** Overdentures, TiZr, Ti Grade IV, SLActive, Split-mouth, Roxolid

## Abstract

**Background:**

The aim of this study was to compare the 5-year survival and success rates of 3.3 mm dental implants either made from titanium-zirconium (TiZr) alloy or from Grade IV titanium (Ti Grade IV) in mandibular implant-based removable overdentures.

**Methods:**

The core study had a follow-up period of 36 months and was designed as a randomized, controlled, double-blind, split-mouth multicenter clinical trial. Patients with edentulous mandibles received two Straumann Bone Level implants (diameter 3.3 mm, SLActive®), one of TiZr (test) and one of Ti Grade IV (control), in the interforaminal region. This follow-up study recruited patients from the core study and evaluated the plaque and sulcus bleeding indices, radiographic crestal bone level, as well as implant survival and success 60 months after implant placement.

**Results:**

Of the 91 patients who initially received implants, 75 completed the 36 month follow-up and 49 were available for the 60 month examination. Two patients were excluded so that a total of 47 patients with an average age of 72 ± 8 years were analysed. The characteristics and 36-month performance of the present study cohort did not differ from the non-included initial participants (*p* > 0.05). In the period since the 36-month follow-up examination, no implant was lost. The cumulative implant survival rate was 98.9 % for the TiZr group and 97.8 % for the Ti Grade IV group. Crestal bone level changes at 60 months were not different in the test and control group (TiZr −0.60 ± 0.69 mm and Ti Grade IV −0.61 ± 0.83 mm; *p* = 0.96). The cumulative implant success rate after 60 months was 95.8 and 92.6 % for TiZr and Ti Grade IV, respectively.

**Conclusions:**

After 60 months, the positive outcomes of the 36 month results for TiZr and Ti Grade IV implants were confirmed, with no significant differences with regard to crestal bone level change, clinical parameters and survival or success rates. TiZr implants performed equally well compared to conventional Ti Grade IV 3.3 mm diameter-reduced implants for mandibular removable overdentures.

**Trial registration:**

Registered on www.clinicaltrials.gov: NCT01878331

## Background

Despite the progress in restorative techniques and preventive measures, tooth loss has still a high prevalence in the older population, yet tends to occur later in life [1–3]. This presents the dental profession with more edentulous patients, where physiological ageing and multimorbidity often dominate the dental treatment planning [4]. Age-adequate treatment planning requires easy to manage and reversible treatment concepts which take into consideration a reduced hand grip strength, dexterity, vision and tactile sensitivity of the patient. Prospective planning is required in view of a potential future functional decline which may render the patient dependent for the activities of daily living. The implant mandibular overdenture with two interforaminal implants presents a multitude of functional and psycho-social improvements for the edentulous patient when compared to a conventional complete denture, which often falls short in fully restoring impaired oral function after tooth loss [5, 6]. Encouraged by long-term success of implant-overdentures, the indications of endosseous implants are more extended to clinically challenging situations in terms of available bone volume for implant anchorage as well as compromised general health conditions [7, 8]. Progress in the implant surfaces have allowed for shorter healing times and improved osseointegration [9]. New alloys have been developed with improved mechanical properties which allow diameter-reduced implants being inserted even in clinically unfavourable anatomical conditions and thus further extend the indications for implant restorations [10]. It is important that implants are designed to have the possibility for a “back-off” strategy if a less retentive and sophisticated restoration is needed in end of life care. In this aspect, two-piece implants present the advantage that the abutment can be removed and exchanged for a lower retention abutment or even a healing cap when functional decline renders denture management or denture wearing difficult. As neuroplasticity is diminished in old age, treatment planning includes avoiding comprehensive changes of dental restorations in very old age, thus preferring versatile and transformable prosthodontic restorations.

Titanium is considered the “gold standard” for dental implants due to its corrosion resistance and biocompatibility [11], but titanium alloys containing zirconium show even better tensile and fatigue strength than pure titanium [12]. For increasing the strength for small-diameter two-piece implants, titanium-zirconium (TiZr) alloy (Roxolid®; Institut Straumann AG, Basel, Switzerland) implants with the SLActive® surface have been introduced. 36 months non-inferiority of Roxolid® implants was reported for mandibular overdentures in a multi-center RCT [13, 14]. The present study aims to confirm the safety and long term clinical performance in terms of crestal bone level change, physical stability and peri-implant health of Roxolid® implants after 60 months in the previously reported patient cohort provided with two-implant-based overdentures.

## Methods

This study was designed as prospective 5 to 10 years follow-up of a randomized, controlled, double-blind, split-mouth, multi-centre clinical trial that came to its end after 36 months (core study). The materials and methods of the core study have been published previously [13, 14] and will be briefly summarized here. The core study has been conducted at eight sites in five countries (Belgium, Germany, Italy, the Netherlands and Switzerland). The follow-up study was conducted at 6 sites in 4 countries (Belgium, Germany, Italy and Switzerland). The study was performed in accordance with the Declaration of Helsinki and Good Clinical Practice (ISO 14155:2011) and approved by the Independent Ethics Committees of the coordinating investigator and all study sites. All participating patients gave their written informed consent. The study was registered at www.clinicaltrials.gov (registration no. NCT01878331 [15]).

### Patients and implants

Patients who had completed the core study were invited to participate in the follow-up study to collect long-term data, 5 and 10 years after implant placement. The patients were selected according to predefined inclusion and exclusion criteria. The inclusion criteria were:

- Treatment in the core study,
- completed 36 month visit of the core study,
- informed and written consent and
- commitment to participate in the study over the entire study duration.

The exclusion criteria were:

- Physical handicaps interfering with the ability to perform adequate oral hygiene,
- failure to attend follow-up visits and
- use of any investigational drug or device during the study period.

During the core study the patients were selected according to predefined inclusion and exclusion criteria. The inclusion criteria were: edentulous mandible, age ≥18 years, last tooth extraction >8 weeks prior to surgery, sufficient bone height of at least 9 mm and bone width for a 3.3 mm diameter implant installation without simultaneous bone augmentation, as well as an edentulous opposing dentition with an implant born or conventional denture or a natural or restored dentition. The exclusion criteria essentially referred to various medical conditions and can be consulted in the first publication of the core study [13]. All patients had presented with an edentulous mandible and had received two Straumann Bone Level implants (Institut Straumann AG, Basel, Switzerland) in the interforaminal region, randomly allocated to one side in a double-blind, split-mouth design. Both implants had exactly the same design with a diameter of 3.3 mm and a SLActive surface, the test implant was fabricated from titanium-zirconium (TiZr) and the control implant from Grade IV titanium (Ti Grade IV).

### Clinical procedure

In the core study, surgery had been performed under local anaesthesia following a standard surgical procedure. Implants of 8, 10, 12 and 14 mm length had been inserted and healing abutments had been installed to allow for trans-mucosal healing. Sutures had been removed 1 to 2 weeks after surgery and the healing abutments had been replaced by Locator abutments (Zest Anchors LLC, Escondido, CA, USA) 6 to 8 weeks after implant placement. Within two weeks following abutment connection the removable dentures were relined to incorporate the female Locator parts. No metal framework was placed. The patients had attended follow-up visits at 6, 12, 24 and 36 months. Patients from 6 centres who consented for the follow-up study were recalled for the 60 month clinical visit. An additional follow-up is planned for 10 years after implant placement.

### Implant survival and success

Implants still in place 60 months after surgery were counted as surviving implants. Adapted from the Buser criteria, implant success was defined as follows: The possibility for restoration, the absence of persistent patient complaints (pain, foreign body sensation and/or dysesthesia), the absence of recurrent peri-implant infection with suppuration, the absence of implant mobility and the absence of continuous radiolucency around the implant [16].

### Peri-implant bone level

**Fig. 1:**
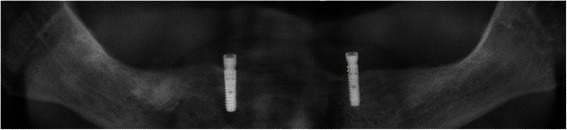
Radiograph showing test and control implant in the interforaminal region. The analysis was performed by an independent investigator using ImageJ software

Standardized panoramic radiographs had been taken at baseline and 6, 12, 24, 36 and 60 months after implant placement (Fig. 1). Film-based images were digitized via video camera, light box and image analysis program [17, 18] and digital images were analyzed using ImageJ 1.33 open software (National Institutes of Health, Bethesda, MD, USA). The analysis of all images was performed by an independent expert.

**Fig. 2:**
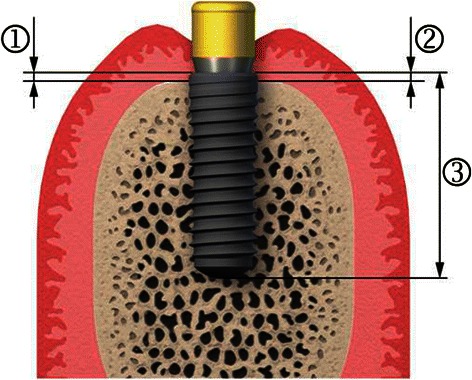
Illustration of the bone level measurements. (1) Chamfer to first implant-to-bone contact, mesial (2) Chamfer to first implant-to-bone contact, distal (3) Length of implant

The known implant length had been used as reference for the analysis. The reference line for the bone level measurements was the implant chamfer 0.2 mm above the implant shoulder. The bone level was defined as distance between the reference point and the first bone-to-implant contact (Fig. 2). The mean value from mesial and distal measurements was used for the evaluation. The bone level change was calculated as a function of the baseline level at implant placement.

### Soft tissue assessment

Soft tissue assessment had been performed at prosthesis placement and 6, 12, 24, 36 and 60 months after implant placement by calibrated operators. Modified Plaque Index (mPI) and the modified Sulcus Bleeding Index (mSBI) according to Mombelli were recorded for the lingual, buccal, mesial and distal sites of the implant [19].

### Safety assessment

Patient safety evaluation included reporting of complications, adverse events (AEs), serious adverse events (SAEs) and device deficiencies. AEs and SAEs were assessed for their relation to the study device and severity.

### Statistical analysis

Efficacy analysis was performed for crestal bone level change, implant survival and success and soft tissue parameters up to 60 months after implant placement based on the “per protocol” (PP) data set. Comparisons between the test and the control group were based on the corresponding 95 % confidence intervals. Changes in crestal bone levels have been compared by t-tests between the treatment groups, the p-values are of descriptive nature. Continuous data are presented as mean values (± standard deviation, SD). For the analysis of crestal bone level changes presented here missing data were not imputed. Differences in the mPI and the mSBI were evaluated by the Wilcoxon Rank Sum test. Kaplan-Meier analysis was used to evaluate implant success and survival and the distributions were compared by log-rank tests. The “safety data” encloses all enrolled patients, who received a study device during the core study. The “intention to treat” (ITT) population comprises all enrolled subjects regardless of any protocol deviation and/or premature termination. The PP population comprises all enrolled patients in whom no major protocol deviation was observed.

## Results

### Patients

**Fig. 3:**
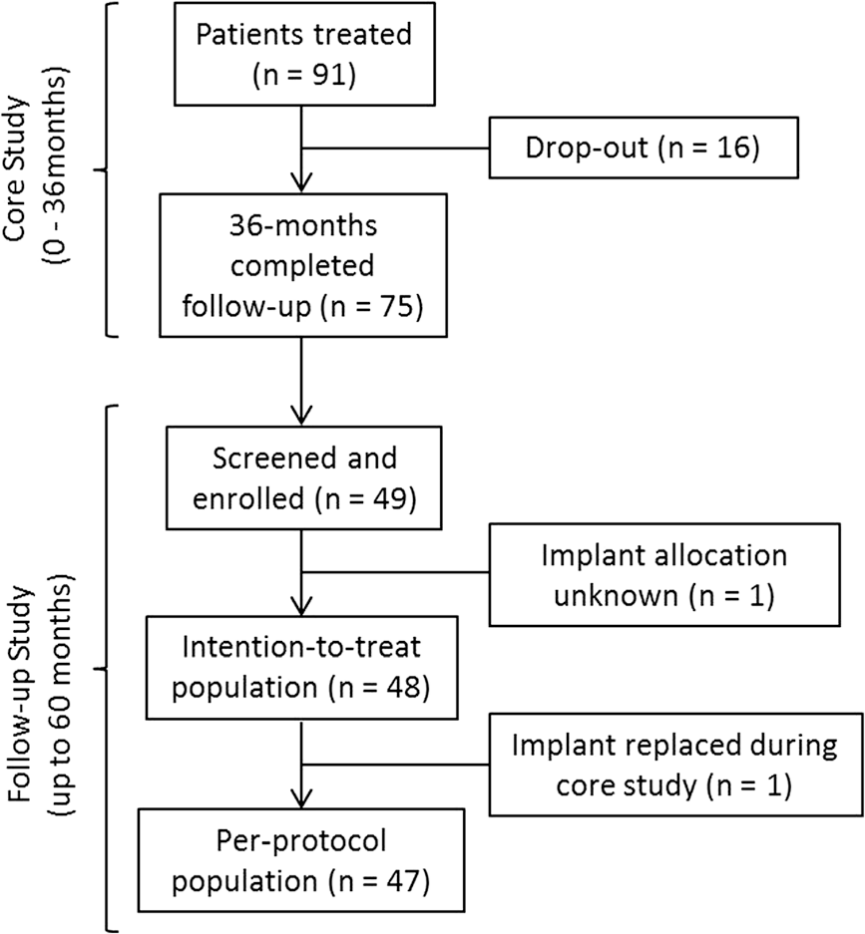
Patient flow diagram for the core- (0 – 36 months) and the follow-up study (up to 60 months)

Ninety-one patients were enrolled in the core study and 75 patients completed the 36 month visit. During this period, 11 patients were lost to follow up, one withdrew consent, one had an adverse event unrelated to the study treatment and three study participants died. Following the 36 month examination, 26 patients were either lost for follow-up or were not eligible for various reasons: One patient was the only patient treated in one centre and it seemed unreasonable to request ethical permission. Another centre did not receive the clearance from the Ethics Committee for the continuation of the study in time; hence further nine patients were not eligible for participation. Finally, 49 patients from the core study were available at the 60 month visit and consented to participate in the follow-up study (Fig. 3). The patient recruitment for the follow-up period started in June 2013 and the last 60 month visit was performed in January 2014.

All 49 patients were eligible for the safety evaluation. One patient was excluded from the ITT population because of unknown implant allocation during the core study (*n* = 48, ITT population). Furthermore, one patient could not be analysed according to the PP population, because one of the two implants was lost and replaced during the core study (*n* = 47, PP population). For many patients time window deviations were observed and categorized as “minor protocol deviations”without further consequences for the data analysis.

The mean age of the PP population was 72 ± 8 years at the 60 month follow-up (range 54 – 92 years). The patient demographic data are presented in Table 1. The majority of patients (87.2 %) suffered from clinically relevant diseases; among the most frequent ones were hypertension and hyperlipidaemia.

**Table 1:** Demographic data of the study population

	Number	Percent
Gender		
Male	24	51.1
Female	23	48.9
Smoking status		
Non-smoker	31	66.0
Past-smoker^a^	16	34.0
Current clinically relevant disease		
Yes^b^	41	87.2
No	6	12.8

Demographic patient data, 60 months after implant placement (PP population, *n* = 47). ^a^ i.e. > 10 cigarettes/day; ^b^ most frequently hypertension and hypercholesterolemia

The primary efficacy variable is implant survival assessed 10 years after implant placement, but the study design includes the assessment of various secondary parameters after 5 and 10 years.

### Implant survival and success

During the observation period of the follow-up study, between 36 and 60 months after implant placement, no implants were lost. However, three implants had been lost during the core study within the first 12 months after implant placement, one in the test group (TiZr) and two in the control group (Ti Grade IV). Kaplan-Meier curves show that the probability of implant survival is declining to 98.9 % for the TiZr group and to 97.8 % for the Ti Grade IV group within the first year after implant placement and remains stable at this level for up to 60 months. There were no significant differences between the two groups (*p* = 0.56). Considering the 26 patients, who were either “lost to follow-up” after completion of the core study or not eligible for participation, as failures, a worst case scenario would result in an implant survival of 53.8 %.

**Fig. 4:**
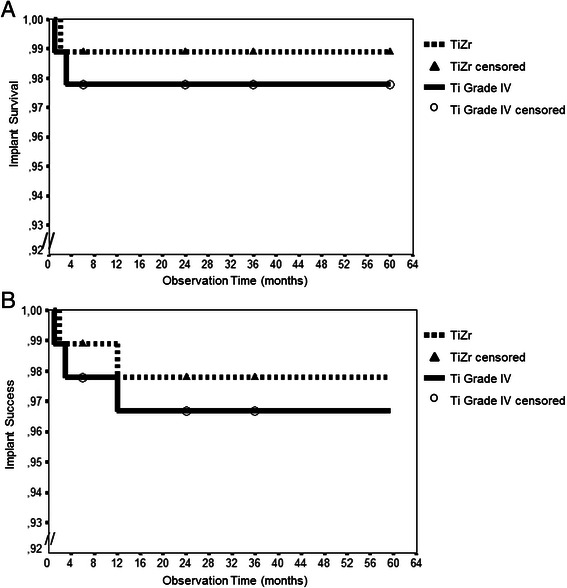
Kaplan-Meier analysis of implant survival and success. **a** Implant survival and **b** success from implant placement to 60 months. Patients lost to follow-up during or after completion of the core study (0–36 months) were censored. Scaling of the Y-axes 0.92 to 1.00

During the core study one implant of each, the test and the control group had been classified as “not successful” due to peri-implant infection and suppuration. In addition, at the 60 month visit one patient showed a peri-implant infection around the TiZr implant. Further, two Ti Grade IV implants were considered not successful due to continuous radiolucency around the implants. Therefore, the cumulative implant success rates (Fig. 4) were 95.8 % and 92.6 % for TiZr and Ti Grade IV, respectively (Kaplan Meier curves; failed implants were also counted as “not successful”; *p* = 0.47).

### Bone level change

**Fig. 5:**
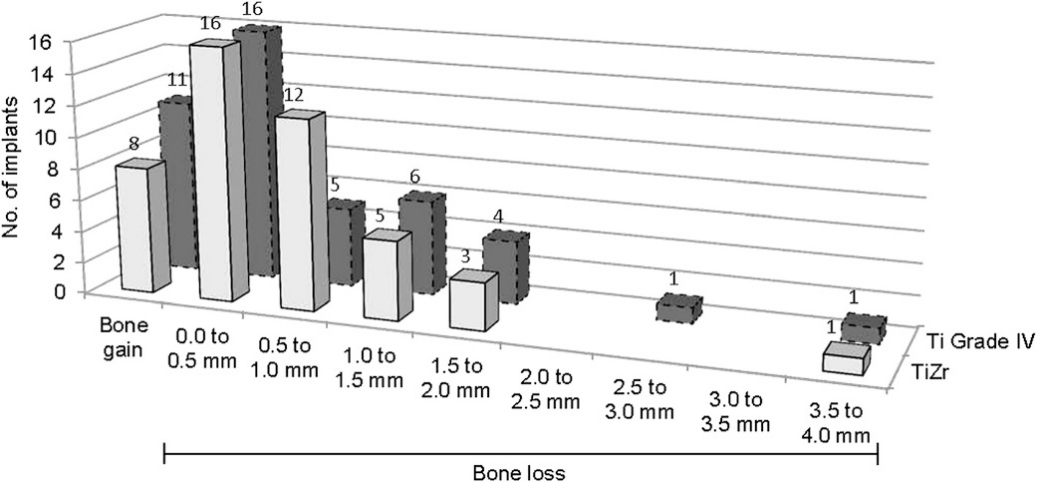
Categorized changes in peri-implant bone level 60 months after implant placement. Implants were categorized according to crestal bone level change (PP population, *n* = 47, some radiographs were impossible to analyse)

**Fig. 6:**
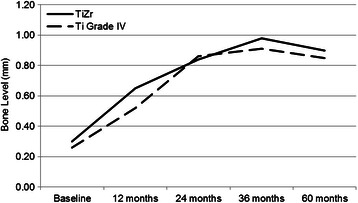
Bone level changes from implant placement to 60 months. Mean peri-implant bone level change up to 60 months (PP population, *n* = 47, some radiographs were impossible to analyse). Positive values: crestal bone level decrease. Negative values: crestal bone level increase. Missing values were excluded from the analysis

There were no significant differences in crestal bone level changes between the TiZr and the Ti Grade IV group, assessed 60 months after implant placement (*p* = 0.96). The mean change in the TiZr group was −0.60 ± 0.69 mm and in the Ti Grade IV group −0.61 ± 0.83 mm, ranging from −3.57 to 0.16 mm and from −3.65 to 0.44 mm, respectively. The majority of implant sites showed crestal bone loss between 0 and 1.0 mm or crestal bone gain (Fig. 5). Crestal bone level changes were more pronounced in the first years after implant placement (Fig. 6). A sensitivity analysis was performed for the core study (12, 24 and 36 months) confirming that there were no significant differences between the original patient population and the population of this follow-up study with regard to crestal bone level changes (*p* = 0.44, 0.41 and 0.61 (Ti Grade IV), respectively *p* = 0.29, 0.35, 0.28 (TiZr), respectively).

### Soft tissue and safety assessments

After 60 months no significant differences in mPI and mSBI were determined between the patients of the TiZr and the Ti Grade IV group (*p* = 0.23 and *p* = 0.77, respectively). Most of the patients showed an mPI score 0 or 1 and the same results were observed for the mSBI (Table 2).

**Table 2:** Plaque index and sulcus bleeding indices after 60 months

	Plaque Index (mPI)		Sulcus Bleeding Index (mSBI)	
	TiZr %	Ti Grade IV %	TiZr %	Ti Grade IV %
Score 0	59.6	68.6	66.5	68.6
Score 1	15.4	16.0	17.0	20.2
Score 2	16.5	8.5	13.8	10.1
Score 3	8.5	7.4	2.7	1.1

Modified Plaque Index and modified Sulcus Bleeding Index according to Mombelli et al. [19], 60 months after implant placement (*p* = 0,23 (mPI), *p* = 0.77 (mSBI), PP population, *n* = 47)

Four of the 49 patients (8.2 %) experienced an AE during the observational period from 36 to 60 months. In accordance with the assessment of implant success, two patients presented with radiolucency around the implant and one patient with peri-implant infection, classified as AEs related to the study device. Another AE, a denture related ulcer, was not related to the implant. None of the patients experienced an SAE between 36 and 60 months after implant placement.

## Discussion

This study was designed as a prospective, randomized, double-blind and split-mouth clinical trial, where dental implants made of TiZr alloy were compared to implants made of Ti Grade IV. Both types of implants had a SLActive surface. After an observation period of 60 months, no significant differences in crestal bone level change, clinical parameters or survival and success rates were found between the groups. The outcomes seen at 12 as well as 36 months continued until 60 months, indicating that TiZr implants in this clinical setting were comparable to Ti Grade IV implants.

Long-term observations are highly relevant when recommending a medical device for clinical use, even more so for elderly patients, where prosthodontic restorations should be designed for long-term survival, as renewal of prostheses might become difficult with increasing frailty and multimorbidity. Adjustments which may become necessary to adapt the prosthodontic restoration to functional decline should rather be performed by a simple alteration of the denture to minimize the challenges to an elderly person’s neuroplasticity and capacity of adaptation. Complications or failures in late life can be minimized when using only well documented and high quality materials for dental restorations. Biological complications may still occur, as the overall risk of implant failure seems influenced by biological parameters like history of periodontal disease or residual periodontal pockets [20–22]. Patient behaviour such as smoking [23, 24] unfavourable oral hygiene [25] or the absence of an adequate peri-implant width of keratinized and attached mucosa [26, 27] may also play a role. Technical aspects such as implant design and surface may also largely vary the clinical outcome, as was recently demonstrated in a large-scale industry independent study on implant survival [28]. In this study the mentioned risk factors were confirmed, and in addition, implant length and implant brand were identified as relevant factors for implant survival and success.

The present data confirm that Roxolid® implants were comparable to the traditional Grade IV titanium alloy in 3.3 mm diameter implants for an implant-supported mandibular overdenture over a 60 months period. This confirmation is of particular importance with regard to the above mentioned concern about safety and quality of implant materials in pre-elderly and elderly patients. The peri-implant bone loss, modified Plaque Index, modified Sulcus Bleeding Index as well as implant success and survival are not statistically different between the two implant materials. One of the strengths of this study is the split-mouth design, which provides an identical biological environment to the test and control implant. Another strength is the use of 3.3 mm diameter implants in a region, where the bone volume might often, but not always be available for larger diameter implants. Even the right- or left handedness of a patient or the preferred chewing side may not have influenced the results, as the side-attribution of the test and control group was randomized. However, any clinical study has inherent inconsistencies, as one patient may vary from the other in a multitude of aspects. A further substantial shortcoming is that not all of the 92 patients who originally received implants were available for all follow-up visits. The core study was planned for 36 months, and ethical permission and insurance had expired after this follow-up period and renewal was necessary. One centre had only one participant recruited, and it seemed unreasonable to undergo the effort of study submission to the Ethics Committee for this single case. A further centre did not obtain ethical approval in time. A total of 26 patients was lost for recruitment for the present study. A worst-case survival rate was therefore calculated at 53.8 %. However, knowing that 10 patients were not included for formal reasons, and taking into account that the included patients did not differ statistically from the not-included participants from the core study at baseline as well as at 12, 24 and 36 month follow-up, it seems reasonable to assume that this worst-case scenario is unrealistic. The crestal bone level changes reported in the present study are within the range reported in the literature for similar clinical indications. A recent meta-analysis on marginal bone level changes at dental implants after an observation period of 60 months concludes that the annual bone loss is below or much below what hitherto has been reported [29]. The marginal bone loss noted in the present patient cohort after 60 months was −0.60 ± 0.69 mm for the TiZr group and −0.61 ± 0.83 mm in the Ti Grade IV group, respectively. The reported bone level changes after 60 months are in between the one reported by Laurell end Lundgren for the Straumann Dental Implant System (0.48 mm (95 % CI −0.598, −0.360) and the Brånemark System with 0.75 mm (95 % CI −0.802, −0.693). When comparing these results with the ones from the meta-analysis, one has to keep in mind that both, elderly and edentulous patients are at particular risk for reduced oral hygiene measures. Around one third of this study’s patient cohort did present with modified Plaque Index and modified Sulcus Bleeding Index scores above zero. However, little is known on the impact of biofilm on the peri-implant bone level [30], especially for elderly patients with an aged immune system. The relation between peri-implantitis and oral hygiene will be of increasing importance for the dental profession, as a growing number of patients with implants will age, hence poor oral hygiene seems pre-programmed.

The benefits of implant overdentures for edentulous patients are well documented and the cost-effectiveness of this treatment protocol has been demonstrated [5, 6, 31]. Compared to conventional dentures, the chewing efficiency may be significantly improved, given that new implant-supported removable overdentures are manufactured [32]. The chewing muscles seem more trained due to the improved chewing performance and after stabilizing a lower denture by means of implants the muscle bulk can be re-gained, even in very old adults [33, 34]. A similar effect of training and re-training was shown also for the leg muscles in a geriatric context [35]. Further improvements of overdentures compared to conventional complete dentures comprise denture satisfaction and Oral Health Related Quality of Life [36], although these outcome measures are complex and may vary between cultures and personalities [37]. Elderly persons are in general less demanding concerning an improvement of their denture performance [38, 39], yet do in general appreciate an improvement of their chewing performance [40–42]. Nevertheless, around one third of edentulous patients reject implant insertion because they object the surgical intervention [43]. Low-diameter implants may not only have a positive effect on the preservation of the residual alveolar ridge and therefore be biologically favourable in certain clinical situations. They may also avoid invasive bone augmentation procedures [44] whereby patient’s morbidity as well as treatment costs and time can be reduced significantly [10] and the smaller the intervention, the more likely is the acceptance in edentulous patients.

## Conclusion

In conclusion it can be stated that the TiZr alloy (Roxolid®) implants provide a long-term safe and reliable alternative to the available portfolio of dental implants, traditionally manufactured from Ti Grade IV. The improved mechanical properties of TiZr may extend the indications in implant therapy to more challenging clinical situations and allow promoting a minimal invasive treatment approach which is particularly suitable for elderly patients.

## Abbreviations

AE: Adverse event
ITT: Intent to treat
mPI: Modified plaque index
mSBI: Modified sulcus bleeding index
PP: Per protocol
SAE: Serious adverse event
SD: Standard deviation
TiZr: Titanium-zirconium
Ti Grade IV: Titanium grade IV
